# Tryptophan and Kynurenine Pathway Metabolites in Animal Models of Retinal and Optic Nerve Damage: Different Dynamics of Changes

**DOI:** 10.3389/fphys.2019.01254

**Published:** 2019-10-01

**Authors:** Michal Fiedorowicz, Tomasz Choragiewicz, Sebastian Thaler, Frank Schuettauf, Dominika Nowakowska, Kamila Wojtunik, Michele Reibaldi, Teresio Avitabile, Tomasz Kocki, Waldemar A. Turski, Agnieszka Kaminska, Pawel Grieb, Eberhart Zrenner, Robert Rejdak, Mario Damiano Toro

**Affiliations:** ^1^Mossakowski Medical Research Centre, Polish Academy of Sciences, Warsaw, Poland; ^2^Department of General Ophthalmology and Pediatric Ophthalmology Service, Medical University of Lublin, Lublin, Poland; ^3^Institute for Ophthalmic Research, University of Tübingen, Tübingen, Germany; ^4^Department of Ophthalmology, University Medical Center, Hamburg-Eppendorf, Hamburg, Germany; ^5^Eye Clinic, University of Catania, Catania, Italy; ^6^Department of Experimental and Clinical Pharmacology, Medical University of Lublin, Lublin, Poland; ^7^Faculty of Medicine, Collegium Medicum, Cardinal Stefan Wyszynski University, Warsaw, Poland

**Keywords:** glaucoma, kynurenine pathway, tryptophan, retina, NMDA, optic nerve crush

## Abstract

Kynurenines, products of tryptophan (TRP) metabolism, display neurotoxic (e.g., 3-hydroxykynurenine; 3-HK), or neuroprotective (e.g., kynurenic acid; KYNA) properties. Imbalance between the enzymes constituting the kynurenine pathway (KP) plays a role in several disease, including neurodegeneration. In this study, we track changes in concentrations of tryptophan and its selected metabolites after damage to retinal ganglion cells and link this data with expression of KP enzymes. Brown-Norway rats were subjected to intravitreal *N*-methyl-D-aspartate (NMDA) injection or partial optic nerve crush (PONC). Retinas were collected 2 and 7 days after the completion of PONC or NMDA injection. Concentrations of TRP, kynurenine (KYN), and KYNA were determined by high performance liquid chromatography (HPLC). Data on gene expression in the rat retina were extracted from GEO, public microarray experiments database. Two days after NMDA injection concentration of TRP decreased, while KYN and KYNA increased. At day 7 compared to day 2 decrease of KYN, KYNA and further reduction of TRP concentration were observed, but on day 7 KYN concentration was still elevated when compared to controls. At day 2 and 7 after NMDA injection no statistically significant alterations of 3-HK were observed. TRP and 3-HK concentration was higher in PONC group than in controls. However, both KYN and KYNA were lower. At day seven concentration of TRP, 3-HK, and KYN was higher, whereas concentration of KYNA declined. *In vivo* experiments showed that retinal damage or optic nerve lesion affect TRP metabolism via KP. However, the pattern of changes in metabolite concentrations was different depending on the model. In particular, in PONC KYNA and KYN levels were decreased and 3-HK elevated. These observations correspond with data on expression of genes encoding KP enzymes assessed after optic nerve crush or transection. After intraorbital optic nerve crush downregulation of *KyatI* and *KyatIII* between 24 h and 3 days after procedure was observed. *Kmo* expression was transiently upregulated (12 h after the procedures). After intraorbital optic nerve transsection (IONT) *Kmo* expression was upregulated after 48 h and 7 days, *KyatI* and *KyatIII* were downregulated after 12, 48 h, 7 days and upregulated after 15 days. Collected data point to the conclusion that development of therapeutic strategies targeting the KP could be beneficial in diseases involving retinal neurodegeneration.

## Introduction

Kynurenine pathway (KP) is the major route for tryptophan (TRP) metabolism in most mammalian tissues. Through this pathway 95% of dietary TRP is transformed to metabolites known as kynurenines (Fujigaki et al., 2017; Sas et al., 2018). Imbalance in KP has been postulated to contribute to pathological mechanisms in several neurological and neurodegenerative diseases like brain ischemia, epilepsy, major depression, Alzheimer’s disease, Huntington’s disease, Parkinson’s disease, multiple sclerosis, HIV associated neurocognitive disorders, or schizophrenia (Maddison and Giorgini, 2015; Fujigaki et al., 2017; Lovelace et al., 2017).

Kynurenine pathway starts with the oxidative cleavage of TRP catalyzed by one of the rate-limiting enzymes: tryptophan 2,3-dioxygenase (TDO), present mostly in liver or indoleamine 2,3-dioxygenase (IDO), present in other tissues including neuronal cells and glial cells, converting TRP to *N*-formylkynurenine (Maddison and Giorgini, 2015). *N*-formylkynurenine is further metabolized by kynurenine formamidase to kynurenine (KYN). KYN is a central metabolite of the KP and its concentration reflects TRP metabolism along the whole KP. It can be metabolized in one of three branches of KP in reactions primarily catalyzed by one of three different enzymes (Fujigaki et al., 2017):

(1) by kynurenine 3-monooxygenase (KMO) to produce 3-hydroxykynurenine (3-HK), subsequently by kynureninase (KYNU) to 3-hydroxyanthranilic acid (3-HAA), then converted to quinolinic acid (QUIN), which is finally converted to NAD^+^;
(2) by one of four kynurenine aminotransferase isoenzymes (KAT I, II, III, IV) to produce kynurenic acid (KYNA) by irreversible transamination of KYN;
(3) or by KYNU to produce anthranilic acid.

Importantly, KP is crucial for cellular energy metabolism through production of nicotinamide adenine dinucleotide (NAD^+^). It is also postulated that KP may modulate immune response through regulation of NAD^+^ availability for energy-demanding immunological reactions (Savitz, 2019).

Several kynurenines display neuroprotective or neurotoxic properties. 3-HK induces oxidative damage and cell death by promoting free radicals production (Okuda et al., 1998; Wei et al., 2000; Ramirez-Ortega et al., 2017). Quinolinic acid (QUIN) is also neurotoxic, possibly acting via activation of glutamatergic *N*-methyl-D-aspartate (NMDA) receptor (Guillemin, 2012). In contrast, KYNA is considered to be neuroprotective. KYNA is the only known endogenous antagonist of the NMDA receptor and non-competitive antagonist of the alpha-7 nicotinic acetylcholine receptor, i.e., KYNA acts as an endogenous modulator of glutamatergic and cholinergic neurotransmission (Sas et al., 2018). It was shown that KYNA can protect neurons against excitotoxic damage caused by QUIN (Ferreira et al., 2018; Pierozan et al., 2018). KYNA is also a ligand for the orphan G protein-coupled receptor (GPR35), which is predominantly located in immune cells, suggesting its role in inflammatory pathogenesis of neurological disorders (Wang et al., 2006). KYNA is an agonist of aryl hydrocarbon receptor (AhR) and functions as a ROS scavenger (Wirthgen et al., 2017).

Presence of KP enzymes and kynurenines was demonstrated in retinas of several vertebrate species (Rejdak et al., 2001, 2003a,b, 2004b). KP was shown to play a role in the retina ontogenesis (Rejdak et al., 2003b). Increase in retinal KYNA content in response to excitotoxic damage was postulated as a part of endogenous anti-excitotoxic defense mechanisms (Rejdak et al., 2003a). KP was also demonstrated to be affected in the course of spontaneous glaucomatous retinal degeneration in DBA/2J mice (Rejdak et al., 2004a; Schuettauf et al., 2007). However, only few TRP metabolites were studied in the retina under pathological conditions.

It has been proposed that increase in retinal KYNA might result either from an enhanced influx of blood-borne KYNA after compromise of the blood-retina barrier or *in situ* increased biosynthesis of KYNA at the lesion site, release from the damaged neurons or from activated microglia and infiltrating macrophages (Lovelace et al., 2017). KP enzymes were shown to be present in the retina: KAT I is preferentially localized on Müller cell endfeet, while KAT II and KAT III are expressed in retinal ganglion cells (Perkins and Stone, 1982; Rejdak et al., 2001, 2003b, 2011). Increased expression of IDO has been shown in retina in the course of diabetic retinopathy, as a result of microglia activation (Hu et al., 2017).

The aim of this study was to track changes in concentrations of tryptophan metabolites in two animal models or retinal degeneration and to link this data with available data on expression of KYNA pathway enzymes.

## Materials and Methods

### Animals

Adult female Brown-Norway rats (Charles River, Sulzfeld, Germany)^1^ with a body weight (BW) of 150–200 g were used. Brown-Norway rats are a pigmented strain that is widely used in experimental ophthalmology. Some recent data show that the outcome of glaucoma-like insult may significantly differ in pigmented and non-pigmented strains (Gurdita et al., 2017). Moreover, albino rats display spontaneous ocular lesions that may interfere with the experimental lesions (Shibuya et al., 2015).

The animals were kept under a 12 h–12 h light–dark cycle with food and water *ad libitum*. All experiments were performed in compliance with the Institute for Laboratory Animal Research [Guide for the Care and Use of Laboratory Animals. Association for Research in Vision and Ophthalmology (ARVO)]. Statement for the Use of Animals in Ophthalmic and Vision Research was also followed. The procedures were approved by respective local ethics committee. Numbers of the tested eyes (*N*) for each experiment are given in the figure captions.

### Intravitreal NMDA Injection

Retinal ganglion cell (RGC) damage was induced by intravitreal NMDA injection as we previously described (Thaler et al., 2010a; Fiedorowicz et al., 2014). Intravitreal NMDA injection induces inner retina damage by overstimulation of NMDA receptors for glutamate (i.e., excitotoxicity), the main excitatory neurotransmitter of the retinal neurons. Briefly, animals were anesthetized by intraperitoneal injection of 7% chloral hydrate solution (6 ml/kg body weight) and local anesthesia in the form of eye drops (oxybuprocaine, Alcaine, Alcon) was also applied. Two microliters of NMDA solution (10 mM in 0.2 phosphate buffered saline, PBS; pH 7.2; reagents obtained from Sigma-Aldrich, Steinheim, Germany)^2^ were intravitreally injected into the posterior side of the globe, 1 mm behind the limbus, with a heat-pulled glass capillary connected to a microsyringe (Drummond Scientific, Broomall, PA, United States)^3^ under direct observation through the microscope. Antibiotic eye drops (ofloxacin, Floxal, Bausch & Lomb, Rochester, NY, United States)^4^ were applied after the injection. Any rat that exhibited lens damage, retinal hemorrhage, retinal detachment, vitreous hemorrhage or other postoperative complications was excluded from the study. Contralateral eyes served as control eyes and were injected with PBS.

### Partial Optic Nerve Crush

Partial optic nerve crush (PONC) was performed as we previously described (Thaler et al., 2010b). Briefly, the animals were anesthetized by intraperitoneal injection of 7% chloral hydrate solution (6 ml/kg body weight). Local anesthesia in the form of eye drops (oxybuprocaine, Alcaine, Alcon, Fort Worth, TX, United States)^5^ was also applied. Optic nerves were exposed by incising the conjunctiva of the eye followed by separation of the retractor bulbi muscle, then piercing and dissecting the meninges with blunt forceps. A cross-action calibrated crush forceps (jaws 0.4 mm apart) was placed approximately 2 mm behind the globe and the nerve was partially crushed for 15 s. Sham operations were similarly performed, but without closing the forceps. In all cases retinal blood supply remained grossly intact, as judged on the basis of a direct microscopic inspection while and after the procedure.

### Sample Collection

Animals were placed in a transparent euthanasia chamber (40 × 30 × 25 cm). CO_2_ (>99.9%) was provided with flow rate at 8 l/min until animals death (CO_2_ gas was maintained for another 2 min after no obvious sigh of breath was observed). Retinas were collected 2 and 7 days after the completion of PONC or NMDA injection. Following hemisection of the eyes along the ora serrata, the cornea, lens and vitreous body were removed. Samples were immediately frozen in liquid nitrogen and stored at −80°C until the biochemical determinations were performed.

### Determination of Tryptophan and Its Metabolites

Tryptophan, KYN, and KYNA concentrations were measured according to Zhao et al. (2010). In brief, studied substances were analyzed by high-performance liquid chromatography (HPLC) system [The UltiMate 3000 Analytical systems (Thermo Fisher Scientific, Waltham, MA, United States)]. The samples were separated on analytical column (Agilent HC-C18; 250 × 4.6 mm, i.d.). The mobile phase was composed of 20 mmol/L NaAc, 3 mmol/L ZnAc_2_, and 7% acetonitrile. It was pumped at a flow-rate of 1 mL/min and the volume per injection was 100 μL. The wavelength of UV detector was set at 365 nm for KYN and at 250 nm for TRP determination. KYNA was quantified fluorometrically (excitation 344 nm, emission 398 nm). 3-hydroxykynurenine (3-HK) was analyzed with the use of an electrochemical detector (The Thermo Scientific Dionex UltiMate 3000 ECD-3000RS), connected to an analytical cell with the oxidation voltage set at 0.6 V, according to the method described by Heyes and Quearry (1988). Waters Spherisorb S3 ODS2 150 × 2.1 mm column (United States) was perfused with a mobile phase consisting of 2% acetonitrile, 0.9% triethylamine, 0.59% phosphoric acid, 0.27 mM sodium EDTA, and 8.9 mM heptane sulfonic acid (flow 0.3 ml/min; the volume per injection was 10 μL). Chromeleon software was used to control HPLC systems and record of chromatographic date. The limit of detection (LOD) was determined on the basis of the calibration curve. LOD of 3-HK was 0.00000248 μg/10 μL. The coefficient variation (CV) in all cases studies did not exceeded threshold of 15–20%.

### Visualization of Retinal Ganglion Cells

Visualization of RGC cells was performed in a separate group of animals as we described previously (Thaler et al., 2012). Briefly, 5 days after PONC/NMDA injection, the animals were anesthetized and 7 μL of (hydroxystilbamidine methanesulfonate, Molecular Probes, Eugene, OR, United States) was applied to both superior colliculi. Two days later, the animals were sacrificed and retinal flat mounts were prepared and evaluated under the microscope.

### Kynurenine Pathway Bioinformatics Gene Expression Analysis

Data on gene expression in the retina was extracted from Gene Expression Omnibus (GEO)^6^ an international public repository of high-throughput functional genomic data sets, with Genevestigator v5.11.05 (Nebion AG, Zurich, Switzerland) using single experiment analysis tool (Hruz et al., 2008). Search phrases were: “Ido,” “Kat,” “Kynu,” “Kmo,” and “*Rattus norvegicus*.” Within found results keywords: “retina” and “RGC” were searched. Only data sets with at least three data points available were included in the analysis. Absolute expression values were plotted using Prism 6 (GraphPad Software, La Jolla, CA, United States).

Our search for expression of genes encoding KP enzymes in the public microarray experiments databases retrieved an *in vitro* study on mechanical stretching of rat Müller cells (Wang et al., 2013) and an *in vivo* study that included intraorbital optic nerve crush (IONC) and intraorbital optic nerve transection (IONT) (Agudo et al., 2008).

### Statistics

Differences in between groups were assessed statistically using the Kruskal–Wallis test followed by Dunn’s multiple comparisons test. *P* < 0.05 was considered statistically significant. Sample sizes (*N*) for each group are provided in the figure captions. Data are presented as mean ± standard deviation.

## Results

**FIGURE 1:**
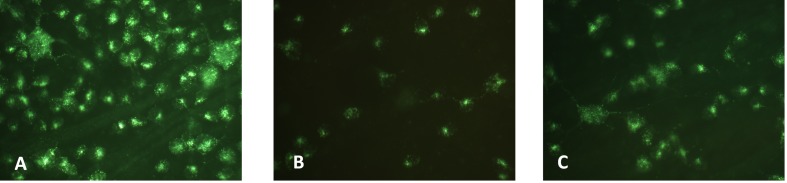
Retinal ganglion cell backlabelling in control retinas from PBS-injected eyes **(A)**, retinas from eyes injected with NMDA **(B)**, or from eyes that underwent PONC **(C)**.

Both NMDA injection and PONC resulted in a remarkable reduction of backlabelled RGCs 7 days after the injury (Figure 1). However, RGC reduction was more pronounced in retinas from NMDA-treated eyes.

**FIGURE 2:**
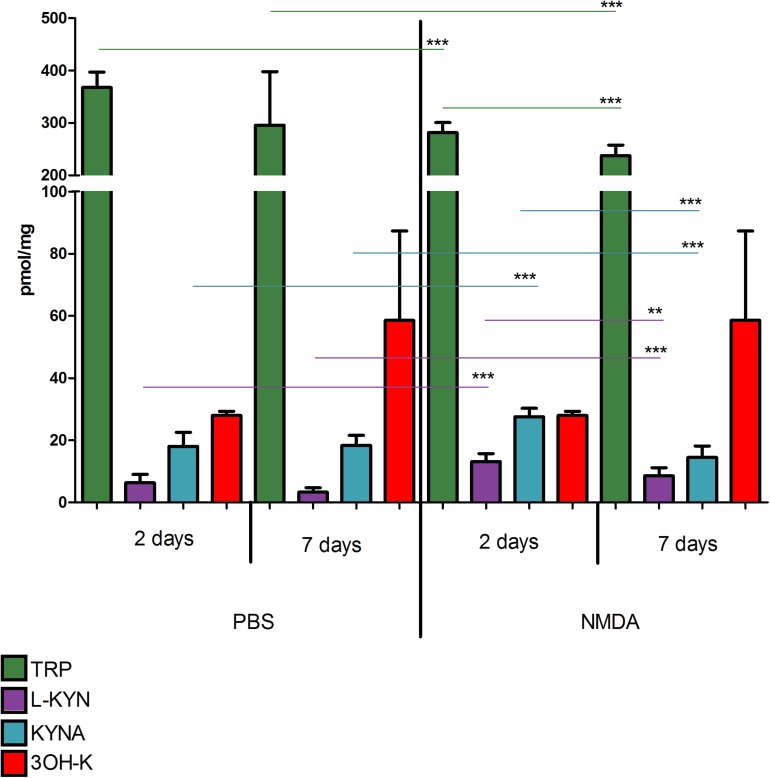
Concentrations of tryptophan (TRP), kynurenine (KYN), kynurenic acid (KYNA), and 3-hydroxykynurenine (3-HK) in retinas of animals after intravitreous injection of NMDA (right side of the graph) or PBS (left side). Kynurenine metabolite concentrations were measured 2 and 7 days after NMDA or PBS injection. *N* = 9, Mean ± SD. ^∗∗^*p* < 0.01, ^∗∗∗^*p* < 0.001.

Concentration of TRP, KYN, 3-HK, and KYNA did not differ significantly in eyes injected with PBS 2 or 7 days before the retinas collection (*p* > 0.05 for all the metabolites, Figure 2). Two days after NMDA injection TRP concentration was lower than in vehicle treated group (281.4 ± 6.5 vs. 367.9 ± 9.7 nmol/g; *p* < 0.0001) but KYN and KYNA levels were higher (KYN 13.13 ± 0.87 vs. 6.32 ± 0.90 nmol/g; *p* < 0.0001; KYNA 27.57 ± 0.91 vs. 18.06 ± 1.50 pmol/g; *p* < 0.0001). Seven days after NMDA injection, concentration of TRP was lower than 2 days after the injection (237.3 ± 6.8 nmol/g, *p* < 0.001; 2 vs. 7 days). Seven days after NMDA injection KYN and KYNA were lower than 2 days after the procedure (KYNA 8.55 ± 0.88 nmol/g, *p* < 0.01 2 vs. 7 days; KYNA 14.50 ± 1.21 pmol/g, *p* < 0.0001; 2 vs. 7 days). However, at day 7 KYN concentration was higher than in vehicle-treated eyes (18.34 ± 1.08 vs. 8.55 ± 0.88 nmol/g; *p* < 0.0001). 3-HK concentration 2 or 7 days after NMDA injection was not significantly different than in PBS-injected eyes (*p* > 0.05).

**FIGURE 3:**
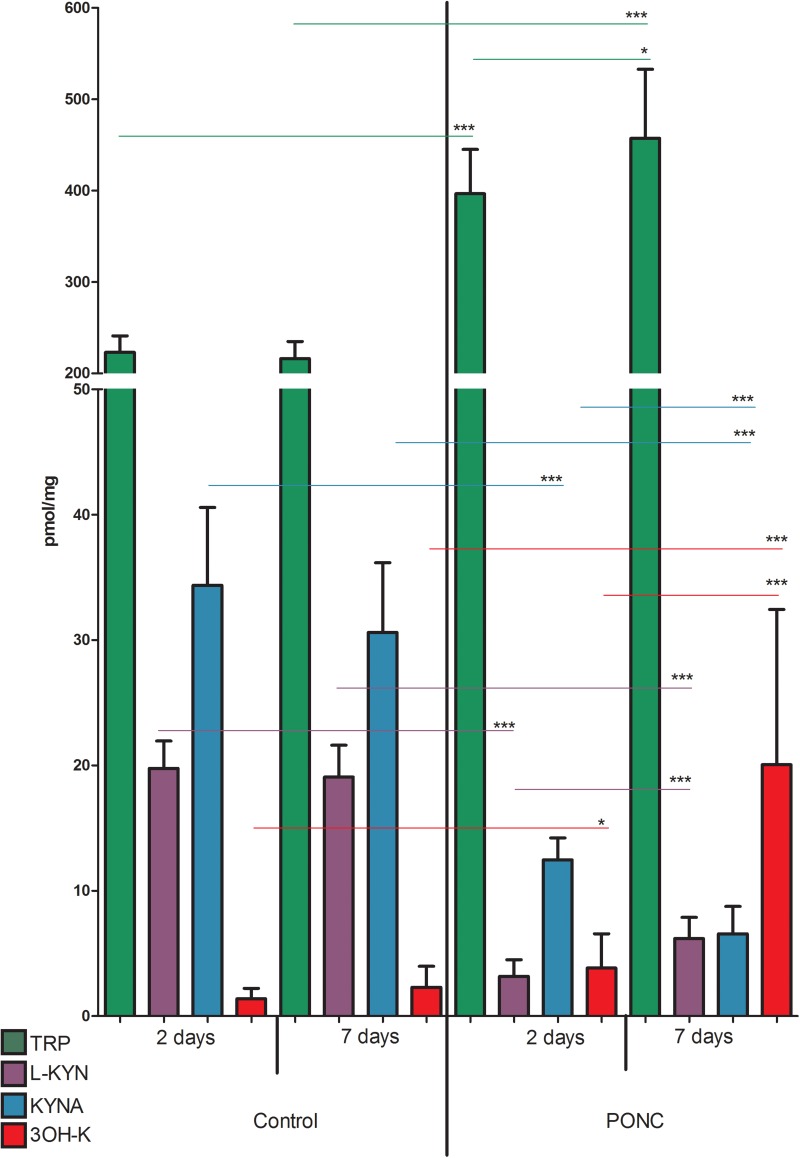
Concentrations of tryptophan (TRP), kynurenine (KYN), kynurenic acid (KYNA), and 3-hydroxykynurenine (3-HK) in retinas of animals that underwent partial optic nerve crush (PONC) (right side of the graph) or sham surgery (left side). Kynurenine metabolite concentrations were measured 2 and 7 days after PONC or sham surgery. *N* = 9 (sham 2 days and PONC 2 days), *N* = 16 (sham 7 days), or *N* = 18 (PONC 7 days). Mean ± SD. ^∗^*p* < 0.05, ^∗∗∗^*p* < 0.001.

Similarly, concentration of TRP, KYN, 3-HK, and KYNA did not differ significantly in animals that underwent sham surgery 2 or 7 days before the retinas collection (*p* > 0.05 for all the metabolites, Figure 3). However, 2 days after PONC procedure TRP and 3-HK concentrations were higher than in sham group (TRP 396.6 ± 16.2 vs. 223.1 ± 6.0 nmol/g, *p* < 0.0001; 3-HK 1.38 ± 0.28 vs. 3.84 ± 0.91 nmol/g, *p* < 0.05) and concentrations of KYN and KYNA were lower than in sham operated animals (KYN 3.17 ± 0.44 vs. 19.76 ± 0.73 nmol/g, *p* < 0.0001; KYNA 12.47 ± 0.58 vs. 34.36 ± 2.07 pmol/g, *p* < 0.0001). Seven days after PONC, concentration of TRP, 3-HK, and KYN was higher than 2 days after the surgery (TRP 457.2 ± 17.8 nmol/g, *p* < 0.05, 2 vs. 7 days; KYN 6.19 ± 0.40 nmol/g, *p* < 0.0001, 2 vs. 7 days; 3-HK 20.06 ± 2.92 nmol/g, *p* < 0.001), whereas concentration of KYNA declined compared to day 2 6.57 ± 0.51 pmol/g, *p* < 0.001, 2 vs. 7 days).

**FIGURE 4:**
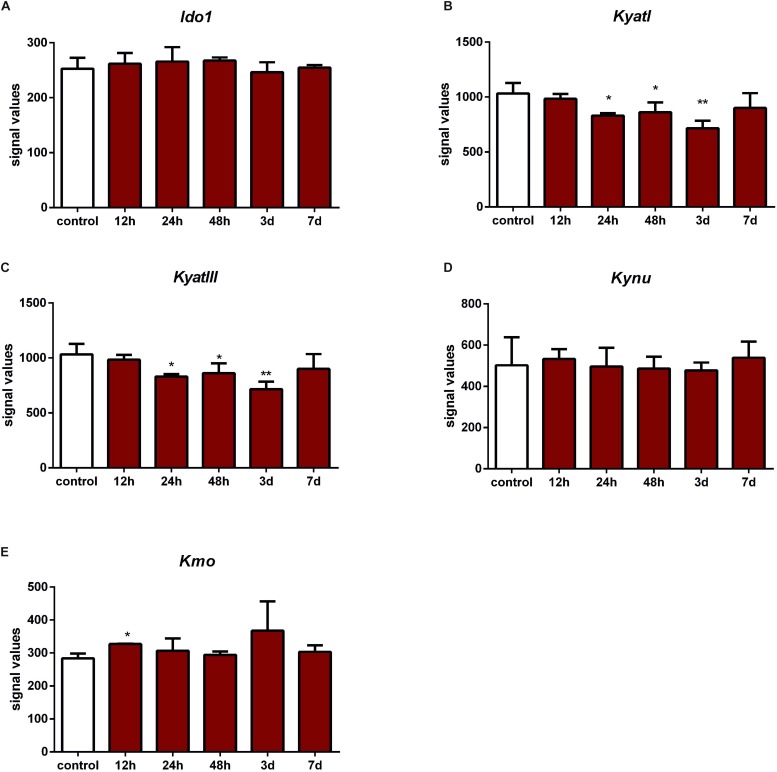
Expression of *KyatI* **(A)**, *KyatIII* **(B)**, *Kynu* **(C)**, *Kmo* **(D)**, and *Kmo* **(E)** in the retinas after intraorbital optic nerve crush. *N* = 5, Mean ± SD. ^∗^*p* < 0.05, ^∗∗^*p* < 0.01.

According to data extracted from GEO, IONC resulted in downregulation of *KyatI* after 24 h (*p* < 0.05), 48 h (*p* < 0.05), and 3 days (*p* < 0.01), and *KyatIII* after 24 h (*p* < 0.05), 48 h (*p* < 0.05), and 3 days (*p* < 0.01). *Kmo* was upregulated only in one time point (12 h, *p* < 0.05). There were no significant changes in expression of *Ido1*, *Kynu*, and *Kmo* (Figure 4).

**FIGURE 5:**
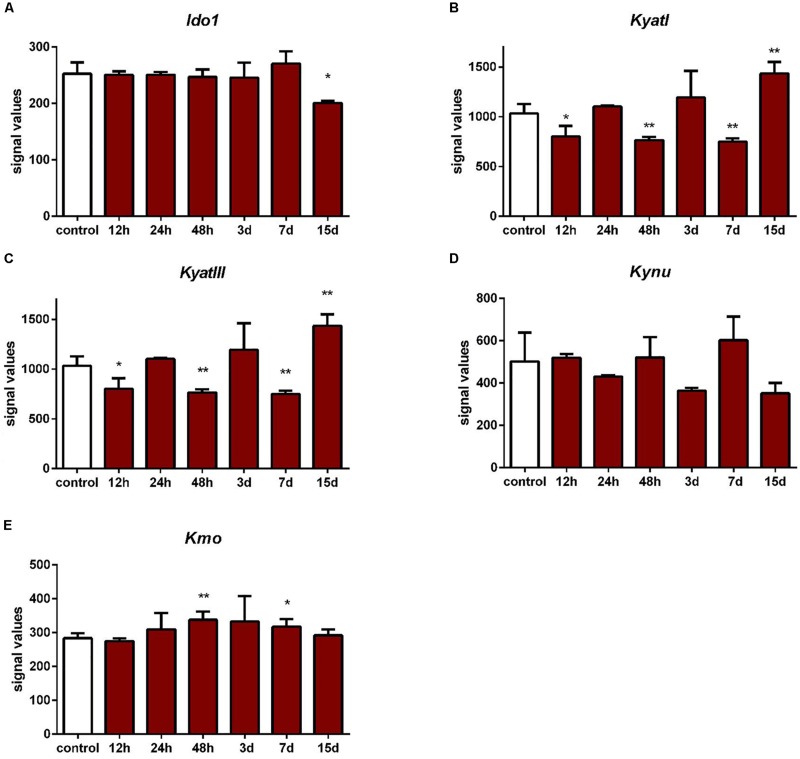
Expression of *Ido1* **(A)**, *KyatI* **(B)**, *KyatIII* **(C)**, *Kynu* **(D)**, and *Kmo* **(E)** in the retinas after intraorbital optic nerve transection. *N* = 3, mean ± SD. ^∗^*p* < 0.05, ^∗∗^*p* < 0.01.

*KyatI* was downregulated after 12 h (*p* < 0.05), 48 h (*p* < 0.01), and 7 days (*p* < 0.01) after IONT. *KyatIII* was downregulated 12 h (*p* < 0.05), 48 h (*p* < 0.01), and 7 days after IONT (*p* < 0.01) and upregulated 15 days after IONT (*p* < 0.01). *Ido1* expression was reduced only 15 days after IONT (*p* < 0.05, Figure 5).

**FIGURE 6:**
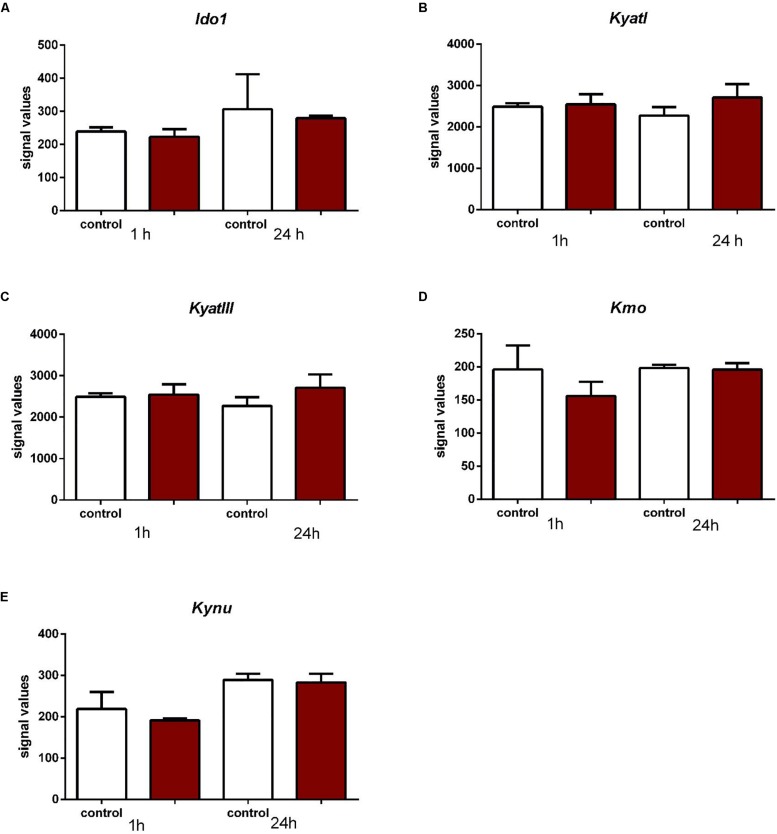
Expression of *Ido1* **(A)**, *KyatI* **(B)**, *KyatIII* **(C)**, *Kynu* **(D)**, and *Kmo* **(E)** in the retinal glial cells exposed to mechanical stretching. *N* = 3, mean ± SD.

In rat Müller cells subjected to stretching forces neither after one or 24 h no changes in expression of *Ido1*, *KyatI*, *KyatIII*, *Kynu*, and *Kmo* in the retinal glial cells exposed to mechanical stretching were observed (Figure 6).

## Discussion

Our experiments demonstrate that concentrations of various TRP metabolites are affected in animal models of retinal damage. However, the pattern of changes in metabolite concentrations was different depending on the model of retinal damage. After NMDA injection TRP concentration declined while KYN level was elevated. KYNA concentration raised transiently 2 days after the insult and then decreased. After PONC TRP and 3-HK concentration was elevated and both KYN and KYNA concentrations decreased. These two models differ in exact mechanisms of damage, its dynamics and severity (Thaler et al., 2010a, 2011).

Mammalian retina seems to be highly susceptible to excitotoxic neurodegeneration. Glutamate neurotoxicity (or excitotoxicity) seems to be an important pathogenetic factor in numerous retinal diseases, e.g., retinal ischemia, glaucoma, retinal detachment, traumatic injuries, diabetic, and high blood pressure retinopathy (Niwa et al., 2016). Excitotoxicity is mediated by excessive activation of glutamate receptors and subsequent calcium flux to the neuronal cells. This phenomenon results in numerous potentially neurotoxic effects: nitric oxide synthase activation, generation of nitric oxide (^∙^NO), activation of phospholipase A2 and excessive production of superoxide radical (O_2_^∙^^–^). ^∙^NO and O_2_^∙^^–^ react and form peroxynitrite (ONOO^–^), a highly nitrating agent that is toxic to neurons (Lipton et al., 1993). Intravitreal administration of NMDA results in a selective RGC death in a dose-dependent manner (Nakazawa et al., 2005). After application of a dose corresponding to our experimental setting, RGC number starts to decrease after 6 h. After 24 h RGC count decrease by over 80% and after 7 days by over 90% (Manabe and Lipton, 2003; Fiedorowicz et al., 2014). After NMDA injection TUNEL-positive cells are observed both in the ganglion cell layer and the inner nuclear layer that contains both neurons (amacrine, horizontal, and bipolar cells) and glial cells (Müller cells) (Manabe and Lipton, 2003). Number of microglia/macrophages in the inner retinal layer was shown to increase 1 day after the NMDA injection and reached its peak 3 days after the procedure (Wada et al., 2013).

Elevation of KYNA after NMDA injection in the current set of experiments correspond to the previously published results (Rejdak et al., 2003a). It seems that elevated KYN and KYNA levels could reflect endogenous anti-excitotoxic defense mechanisms (Rejdak et al., 2003a; Schuettauf et al., 2007; Zarnowski et al., 2007). These changes in KP might correspond to the increase in number of microglia/macrophages cells (Wada et al., 2013). Moreover, a decrease in TRP levels also supports the hypothesis that enhanced *in situ* KYNA synthesis is responsible for its elevation in this model. Unfortunately, there were no available data on KP enzymes gene expression after intravitreous NMDA administration.

Partial optic nerve crush is more complex model of retinal damage than NMDA injection. The injury and the cascade of secondary events reflects the pathological changes occurring in traumatic optic neuropathy and mimics some features of glaucoma (Yoles and Schwartz, 1998). Extent of RGC loss after the optic nerve crush depends on duration of the procedure (Tan et al., 2012). In our setting RGC count is reduced by over 50% 7 days after PONC as we shown in our previous works (Schuettauf et al., 2000; Thaler et al., 2011).

**FIGURE 7:**
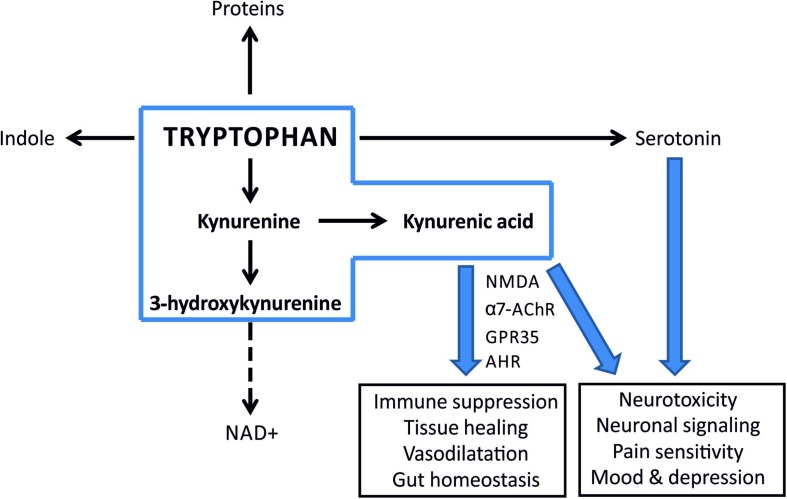
A simplified scheme illustrating metabolic fates of tryptophan and potential consequences of imbalance in its turnover. Kynurenine pathway is the major route for tryptophan metabolism in most mammalian tissues (the metabolites within the scope of this paper are inside a blue box). KYNA is an agonist of aryl hydrocarbon receptor (AhR) and functions as an ROS scavenger and suppresses inflammatory response. A shift toward 3-HK and NAD^+^ production enhances inflammation.

Despite the increased TRP levels after PONC, we noted a decrease in both KYN and KYNA levels. These observations correspond with gene expression data after both after optic nerve crush and transection, where *Ido1* and *Kynu* expression is unchanged, *Kmo* elevated, *KyatI* and *KyatIII* reduced. It suggests that in this case the TRP metabolism through KP is shifted toward production of toxic downstream KYN metabolites that could contribute to the retinal damage in this model (Figure 7). This supposition is supported with the observation that a neurotoxic 3-HK metabolite was significantly elevated in this model. KMO, an enzyme catalyzing KYN conversion to 3-HK is primarily produced in microglia in the nervous system (Parrott and O’Connor, 2015). Therefore, a shift toward “neurotoxic” 3-HK branch of KP may indicate microglia activation in PONC. This is in agreement with previous results showing that in optic nerve crush opening of the blood-brain/retina barrier and subsequent massive microglia/macrophages flux occurs. Microglia/macrophages were detected by immunohistochemistry at the lesion site 2 days after crush and the number of these cells peaked 6 days after crush (Thaler et al., 2011). Data obtained in ocular tissue correspond with that derived from central nervous system cells, where glial origin KAT presented on proliferating (astro)glial cells in lesion site is responsible for increased KYNA production (Wu et al., 1991).

An interesting new direction for the evaluation of the role on KP imbalance in retinal neurodegeneration would be identification of cells involved in the observed phenomena in two models of retinal and optic nerve damage. Another open important question is whether KP modulation in NMDA-induced retinal toxicity or PONC will enable inhibition of retinal damage. Our study on effects of acetoacetate and β-hydroxybutyrate administration in NMDA-induced toxicity model suggest that KP modulation may be involved in the neuroprotective mechanism of this approach (Thaler et al., 2010a). Another work showed that systemic administration of KYNA precursor KYN prevents NMDA-induced retinal damage (Vorwerk et al., 1996). It is therefore probable that other neuroprotective approaches are also at least partially mediated by KP normalization that could be an attractive target for neuroprotection of the retina.

A limitation of our study is mRNA analysis based on public GEO database and not on our samples. However, this approach is recently quite frequently used (Yang and Mei, 2015; Cui et al., 2018; Giummarra et al., 2018). Another limitation of our study design is a lack of parallel analysis of inflammatory markers in the current study. Since the amount of retinal tissue is very limited, we decided to perform extensive chromatographic determinations of kynurenines to meet the main goal of the study. It is noteworthy that the inflammatory aspects of retinal and optic nerve damage in both utilized models were described previously (Thaler et al., 2011).

## Conclusion

Our results demonstrate imbalance in KP in the insulted retina. The KP response to retinal/optic nerve damage depended on the nature of the insult. In particular mechanical damage to the optic nerve resulted in remarkable decline in retinal KYNA concentration and this response could be explained by differences in the expression of KP enzymes. Our results support the view that development of different strategies targeting the KP and leading to increased KYNA concentration could be beneficial in diseases involving retinal neurodegeneration. Future studies are needed to fully elucidate contribution of KP enzymes to retinal degeneration and its potential for future neuroprotective therapies.

## Data Availability Statement

All datasets generated for this study are included in the manuscript/supplementary files.

## Ethics Statement

The animal study was reviewed and approved by the Ethics Committee of the Medical University of Lublin.

## Author Contributions

MT and RR conceived and designed the study and administered the project as Principal Investigators. MF and TC wrote the first draft of the manuscript. MF, TC, ST, and FS performed the animal experiments. TK and WT performed the biochemical determinations. DN, KW, and MR curated and analyzed the data. MT, AK, WT, PG, EZ, and TA revised and validated the manuscript. All authors gave final approval of the submitted version.

## Conflict of Interest

The authors declare that the research was conducted in the absence of any commercial or financial relationships that could be construed as a potential conflict of interest.

## Footnotes

1 https://www.criver.com/

2 https://www.sigmaaldrich.com/

3 https://www.drummondsci.com/

4 https://www.bausch.com/

5 https://www.alcon.com/

6 https://www.ncbi.nlm.nih.gov/geo/

## References

[B1] Agudo M. Perez-Marin M. C. Lonngren U. Sobrado P. Conesa A. Canovas I. (2008). Time course profiling of the retinal transcriptome after optic nerve transection and optic nerve crush. *Mol. Vis.* 14 1050–1063. 18552980 PMC2426719

[B2] Cui Z. Zeng Q. Guo Y. Liu S. Chen J. (2018). Integrated bioinformatic changes and analysis of retina with time in diabetic rats. *PeerJ* 6:e4762. 10.7717/peerj.4762 29785346 PMC5960260

[B3] Ferreira F. S. Biasibetti-Brendler H. Pierozan P. Schmitz F. Berto C. G. Prezzi C. A. (2018). Kynurenic acid restores Nrf2 levels and prevents quinolinic acid-induced toxicity in rat striatal slices. *Mol. Neurobiol.* 55 8538–8549. 29564809 10.1007/s12035-018-1003-2

[B4] Fiedorowicz M. Rejdak R. Schuettauf F. Wozniak M. Grieb P. Thaler S. (2014). Age-dependent neuroprotection of retinal ganglion cells by tempol-C8 acyl ester in a rat NMDA toxicity model. *Folia Neuropathol.* 52 291–297. 10.5114/fn.2014.45570 25310740

[B5] Fujigaki H. Yamamoto Y. Saito K. (2017). L-tryptophan-kynurenine pathway enzymes are therapeutic target for neuropsychiatric diseases: focus on cell type differences. *Neuropharmacology* 112(Pt B), 264–274. 10.1016/j.neuropharm.2016.01.011 26767951

[B6] Giummarra L. Crewther S. G. Riddell N. Murphy M. J. Crewther D. P. (2018). Pathway analysis identifies altered mitochondrial metabolism, neurotransmission, structural pathways and complement cascade in retina/RPE/choroid in chick model of form-deprivation myopia. *PeerJ* 6:e5048. 10.7717/peerj.5048 29967729 PMC6026464

[B7] Guillemin G. J. (2012). Quinolinic acid: neurotoxicity. *FEBS J.* 279:1355. 10.1111/j.1742-4658.2012.08493.x 22251552

[B8] Gurdita A. Tan B. Joos K. M. Bizheva K. Choh V. (2017). Pigmented and albino rats differ in their responses to moderate, acute and reversible intraocular pressure elevation. *Doc. Ophthalmol.* 134 205–219. 10.1007/s10633-017-9586-x 28389912 PMC5545899

[B9] Heyes M. P. Quearry B. J. (1988). Quantification of 3-hydroxykynurenine in brain byhigh- performance liquid chromatography and electrochemical detection. *J. Chromatogr.* 428 340–344. 10.1016/s0378-4347(00)83925-0 3215936

[B10] Hruz T. Laule O. Szabo G. Wessendorp F. Bleuler S. Oertle L. (2008). Genevestigator v3: a reference expression database for the meta-analysis of transcriptomes. *Adv. Bioinform.* 2008:420747. 10.1155/2008/420747 19956698 PMC2777001

[B11] Hu P. Hunt N. H. Arfuso F. Shaw L. C. Uddin M. N. Zhu M. (2017). Increased indoleamine 2,3-dioxygenase and quinolinic acid expression in microglia and muller cells of diabetic human and rodent retina. *Invest. Ophthalmol. Vis. Sci.* 58 5043–5055. 10.1167/iovs.17-21654 28980000 PMC5633007

[B12] Lipton S. A. Choi Y. B. Pan Z. H. Lei S. Z. Chen H. S. Sucher N. J. (1993). A redox-based mechanism for the neuroprotective and neurodestructive effects of nitric oxide and related nitroso-compounds. *Nature* 364 626–632. 10.1038/364626a0 8394509

[B13] Lovelace M. D. Varney B. Sundaram G. Lennon M. J. Lim C. K. Jacobs K. (2017). Recent evidence for an expanded role of the kynurenine pathway of tryptophan metabolism in neurological diseases. *Neuropharmacology* 112(Pt B), 373–388. 10.1016/j.neuropharm.2016.03.024 26995730

[B14] Maddison D. C. Giorgini F. (2015). The kynurenine pathway and neurodegenerative disease. *Semin. Cell Dev. Biol.* 40 134–141. 10.1016/j.semcdb.2015.03.002 25773161

[B15] Manabe S. Lipton S. A. (2003). Divergent NMDA signals leading to proapoptotic and antiapoptotic pathways in the rat retina. *Invest. Ophthalmol. Vis. Sci.* 44 385–392. 12506100 10.1167/iovs.02-0187

[B16] Nakazawa T. Shimura M. Endo S. Takahashi H. Mori N. Tamai M. (2005). N-methyl-D-Aspartic acid suppresses akt activity through protein phosphatase in retinal ganglion cells. *Mol. Vis.* 11 1173–1182. 16402025

[B17] Niwa M. Aoki H. Hirata A. Tomita H. Green P. G. Hara A. (2016). Retinal cell degeneration in animal models. *Int. J. Mol. Sci.* 17:E110. 10.3390/ijms17010110 26784179 PMC4730351

[B18] Okuda S. Nishiyama N. Saito H. Katsuki H. (1998). 3-Hydroxykynurenine, an endogenous oxidative stress generator, causes neuronal cell death with apoptotic features and region selectivity. *J. Neurochem.* 70 299–307. 10.1046/j.1471-4159.1998.70010299.x 9422375

[B19] Parrott J. M. O’Connor J. C. (2015). Kynurenine 3-monooxygenase: an influential mediator of neuropathology. *Front. Psychiatry* 6:116. 10.3389/fpsyt.2015.00116 26347662 PMC4542134

[B20] Perkins M. N. Stone T. W. (1982). An iontophoretic investigation of the actions of convulsant kynurenines and their interaction with the endogenous excitant quinolinic acid. *Brain Res.* 247 184–187. 10.1016/0006-8993(82)91048-4 6215086

[B21] Pierozan P. Biasibetti-Brendler H. Schmitz F. Ferreira F. Pessoa-Pureur R. Wyse A. T. S. (2018). Kynurenic acid prevents cytoskeletal disorganization induced by quinolinic acid in mixed cultures of rat striatum. *Mol. Neurobiol.* 55 5111–5124. 28840509 10.1007/s12035-017-0749-2

[B22] Ramirez-Ortega D. Ramiro-Salazar A. Gonzalez-Esquivel D. Rios C. Pineda B. Perez de la Cruz V. (2017). 3-hydroxykynurenine and 3-hydroxyanthranilic acid enhance the toxicity induced by copper in rat astrocyte culture. *Oxid. Med. Cell Longev.* 2017:2371895. 10.1155/2017/2371895 28831293 PMC5555010

[B23] Rejdak R. Kohler K. Kocki T. Shenk Y. Turski W. A. Okuno E. (2004a). Age-dependent decrease of retinal kynurenate and kynurenine aminotransferases in DBA/2J mice, a model of ocular hypertension. *Vis. Res.* 44 655–660. 10.1016/j.visres.2003.11.003 14751550

[B24] Rejdak R. Shenk Y. Schuettauf F. Turski W. A. Okuno E. Zagorski Z. (2004b). Expression of kynurenine aminotransferases in the rat retina during development. *Vis. Res.* 44 1–7. 10.1016/j.visres.2003.07.007 14599566

[B25] Rejdak R. Rummelt C. Zrenner E. Grieb P. Rejdak K. Okuno E. (2011). Presence of L-kynurenine aminotransferase III in retinal ganglion cells and corpora amylacea in the human retina and optic nerve. *Folia Neuropathol.* 49 132–137. 21845542

[B26] Rejdak R. Zarnowski T. Turski W. A. Kocki T. Zagorski Z. Zrenner E. (2003a). Alterations of kynurenic acid content in the retina in response to retinal ganglion cell damage. *Vis. Res.* 43 497–503. 10.1016/s0042-6989(02)00682-x 12594996

[B27] Rejdak R. Zielinska E. Shenk Y. Turski W. A. Okuno E. Zarnowski T. (2003b). Ontogenic changes of kynurenine aminotransferase I activity and its expression in the chicken retina. *Vis. Res.* 43 1513–1517. 10.1016/s0042-6989(03)00233-5 12782065

[B28] Rejdak R. Zarnowski T. Turski W. A. Okuno E. Kocki T. Zagorski Z. (2001). Presence of kynurenic acid and kynurenine aminotransferases in the inner retina. *Neuroreport* 12 3675–3678. 10.1097/00001756-200112040-00014 11726772

[B29] Sas K. Szabo E. Vecsei L. (2018). Mitochondria, oxidative stress and the kynurenine system, with a focus on ageing and neuroprotection. *Molecules* 23:E191. 10.3390/molecules23010191 29342113 PMC6017505

[B30] Savitz J. (2019). The kynurenine pathway: a finger in every pie. *Mol. Psychiatry* 10.1038/s41380-019-0414-414 [Epub ahead of print]. 30980044 PMC6790159

[B31] Schuettauf F. Naskar R. Vorwerk C. K. Zurakowski D. Dreyer E. B. (2000). Ganglion cell loss after optic nerve crush mediated through AMPA-kainate and NMDA receptors. *Invest. Ophthalmol. Vis. Sci.* 41 4313–4316. 11095632

[B32] Schuettauf F. Thaler S. Bolz S. Fries J. Kalbacher H. Mankowska A. (2007). Alterations of amino acids and glutamate transport in the DBA/2J mouse retina; possible clues to degeneration. *Graefes Arch. Clin. Exp. Ophthalmol.* 245 1157–1168. 10.1007/s00417-006-0531-z 17226020

[B33] Shibuya K. Tomohiro M. Sasaki S. Otake S. (2015). Characteristics of structures and lesions of the eye in laboratory animals used in toxicity studies. *J. Toxicol. Pathol.* 28 181–188. 10.1293/tox.2015-2037 26538807 PMC4604127

[B34] Tan H. B. Shen X. Cheng Y. Jiao Q. Yang Z. J. Zhong Y. S. (2012). Evaluation of a partial optic nerve crush model in rats. *Exp. Ther. Med.* 4 401–404. 10.3892/etm.2012.619 23181107 PMC3503534

[B35] Thaler S. Choragiewicz T. J. Rejdak R. Fiedorowicz M. Turski W. A. Tulidowicz-Bielak M. (2010a). Neuroprotection by acetoacetate and beta-hydroxybutyrate against NMDA-induced RGC damage in rat–possible involvement of kynurenic acid. *Graefes Arch. Clin. Exp. Ophthalmol.* 248 1729–1735. 20532550 10.1007/s00417-010-1425-7 PMC2974203

[B36] Thaler S. Fiedorowicz M. Rejdak R. Choragiewicz T. J. Sulejczak D. Stopa P. (2010b). Neuroprotective effects of tempol on retinal ganglion cells in a partial optic nerve crush rat model with and without iron load. *Exp. Eye Res.* 90 254–260. 10.1016/j.exer.2009.10.013 19883642

[B37] Thaler S. Fiedorowicz M. Grieb P. Wypych Z. Knap N. Borowik T. (2011). Neuroprotective effects of tempol acyl esters against retinal ganglion cell death in a rat partial optic nerve crush model. *Acta Ophthalmol.* 89:e555-60. 10.1111/j.1755-3768.2011.02180.x 21645284

[B38] Thaler S. Voykov B. Willmann G. Fiedorowicz M. Rejdak R. Gekeler F. (2012). Tempol protects against intravitreous indocyanine green-induced retinal damage in rats. *Graefes Arch. Clin. Exp. Ophthalmol.* 250 1597–606. 10.1007/s00417-012-2000-1 22460632

[B39] Vorwerk C. K. Kreutz M. R. Dreyer E. B. Sabel B. A. (1996). Systemic L-kynurenine administration partially protects against NMDA, but not kainate-induced degeneration of retinal ganglion cells, and reduces visual discrimination deficits in adults rats. *Invest. Ophthalmol. Vis. Sci.* 37 2382–2392. 8933755

[B40] Wada Y. Nakamachi T. Endo K. Seki T. Ohtaki H. Tsuchikawa D. (2013). PACAP attenuates NMDA-induced retinal damage in association with modulation of the microglia/macrophage status into an acquired deactivation subtype. *J. Mol. Neurosci.* 51 493–502. 23720065 10.1007/s12031-013-0017-5

[B41] Wang J. Simonavicius N. Wu X. Swaminath G. Reagan J. Tian H. (2006). Kynurenic acid as a ligand for orphan G protein-coupled receptor GPR35. *J. Biol. Chem.* 281 22021–22028. 10.1074/jbc.M603503200 16754668

[B42] Wang X. Fan J. Zhang M. Sun Z. Xu G. (2013). Gene expression changes under cyclic mechanical stretching in rat retinal glial (Muller) cells. *PLoS One* 8:e63467. 10.1371/journal.pone.0063467 23723984 PMC3664568

[B43] Wei H. Leeds P. Chen R. W. Wei W. Leng Y. Bredesen D. E. (2000). Neuronal apoptosis induced by pharmacological concentrations of 3-hydroxykynurenine: characterization and protection by dantrolene and Bcl-2 overexpression. *J. Neurochem.* 75 81–90. 10.1046/j.1471-4159.2000.0750081.x 10854250

[B44] Wirthgen E. Hoeflich A. Rebl A. Gunther J. (2017). Kynurenic acid: the janus-faced role of an immunomodulatory tryptophan metabolite and its link to pathological conditions. *Front. Immunol.* 8:1957. 10.3389/fimmu.2017.01957 29379504 PMC5770815

[B45] Wu H. Q. Turski W. A. Undgerstedt U. Schwarcz R. (1991). Systemic kainic acid administration in rats: effects on kynurenic acid production in vitro and in vivo. *Exp. Neurol.* 113 47–52. 10.1016/0014-4886(91)90145-3 2044678

[B46] Yang Y. Mei Q. (2015). miRNA signature identification of retinoblastoma and the correlations between differentially expressed miRNAs during retinoblastoma progression. *Mol. Vis.* 21 1307–1317. 26730174 PMC4688417

[B47] Yoles E. Schwartz M. (1998). Elevation of intraocular glutamate levels in rats with partial lesion of the optic nerve. *Arch. Ophthalmol.* 116 906–910. 9682704 10.1001/archopht.116.7.906

[B48] Zarnowski T. Rejdak R. Zielinska-Rzecka E. Zrenner E. Grieb P. Zagorski Z. (2007). Elevated concentrations of kynurenic acid, a tryptophan derivative, in dense nuclear cataracts. *Curr. Eye Res.* 32 27–32. 10.1080/02713680601090965 17364732

[B49] Zhao J. Gao P. Zhu D. (2010). Optimization of Zn2+-containing mobile phase for simultaneous determination of kynurenine, kynurenic acid and tryptophan in human plasma by high performance liquid chromatography. *J. Chromatogr. B Analyt. Technol. Biomed. Life Sci.* 878 603–608. 10.1016/j.jchromb.2010.01.006 20102795

